# Cancer of the Neck of the Womb Treated by Scraping—Douglas’s Cul-de-sac Opened—Recovery without a Bad Symptom—Abdominal Enlargement Probably Due to Hysteria*Specially Reported for the Buffalo Medical and Surgical Journal.

**Published:** 1883-02

**Authors:** William Goodell

**Affiliations:** Professor of Clinical Gynecology in the University of Pennsylvania


### BUFFALO

### Medical and Surgical Journal.

Vol. XXII. FEBRUARY, 1883. No. 7.

### Original Communications.

### Cancer of the Neck of the Womb Treated by Scraping—Douglas's Cul-de-sac Opened—Recovery without a Bad Symptom—Abdominal Enlargement Probably due to Hysteria.*

A CLINICAL LECTURE DELIVERED AT THE HOSPITAL OF THE UNIVERSITY OF PENNSYLVANIA, NOV. 1, 1882.

By William Goodell, M. D.

Professor of Clinical Gynecology in the University of Pennsylvania.

Gentlemen—The history of this case alone will, I think, enable you to make the diagnosis. It is as follows: She is forty-two years of age, has had eight children, the youngest of which is two years of age. She has done her duty to the commonwealth, and in these days when there is often but one child in twenty years, a woman who has had eight children is worthy of a civic crown. Her labors were so easy that a physician was never called to any of them. During the past year she has had a very offensive discharge. The menses were regular until two or three months ago, when the discharge appeared every two weeks, for two or three times, but there was an in-

* Specially Reported for the Buffalo Medical and Surgical Journal.

terval of a month between the last periods, which were normal in quantity. She complains of dull pain, and occasional shooting pains in the back.

What is the significance of an offensive discharge in a woman who has borne children? The first thing you think of is carcinoma, but the discharge might be due to something else. It might be produced by a fibroid tumor, which had become necrosed, but such a tumor would cause severe hemorrhage, metrorrhagia and menorrhagia. She has not had these. We may, therefore, put aside the idea of fibroid tumor.

Why would our first thought be of carcinoma? I have told you on previous occasions that this is not a disease of virgins and sterile women. It is rare to find it in women who have not borne children. I have never seen it in such a case but once, and even then I should not be willing to swear that the woman had never borne a child. I believe the reason that carcinoma of the neck ot the womb is almost exclusively found in those who have born children, is because it has its seat in a laceration of the cervix. The laceration does not heal; the delicate epithelium is exposed to abrasion from coition and to abrasion from locomotion; the denuded surface is kept raw, and all raw surfaces are liable to degenerate.

How many kinds of cancer are there and how shall we treat them? Are we, so to speak, localists or constitutionalists? There are three varieties of cancer which may attack the neck of the womb, but I believe them to be different expressions of the same form of disease. In the first stage we have scirrhus, in the second we have epithelioma, and in the third (which we rarely see) encephaloid.

Epithelioma is the variety which is most frequently met with. It occurs in two forms, the excavating ulcer and the vegetating. The former is the most common. In it the cervix is found enlarged from the deposit of inflammatory matters which may or may not be of a cancerous nature. In the centre we find, usually, an excavated sore resembling the crater of a volcano, surrounded by a hard margin and containing friable granulations which bleed on the slightest provocation.

Is this a local or constitutional disease? Under what banner shall we range ourselves? If we accept the opinion that it is a local disease, we may accomplish great good, but if we consider it to be constitutional, we may as well sit down and fold our hands, for we can not hope to effect any improvement by local measures. My own conviction, or rather, let me say, my own impression is, that cancer is at first a local disease, and that as the disease continues, it becomes constitutional. My reason for this opinion is that in three cases I have accomplished an absolute cure by local means. In other cases the disease has been kept in abeyance, and in still other instances, it has never returned in the cervix after having been once removed, but has attacked some other organ. As I have said, my opinion is that it starts as a local disease, and if we see the case in the early stage, we may hope to effect a cure, which in a later stage, after the disease has become constitutional, is impossible.

There is a wide-spread error against which I desire to warn you. In thinking of cancer, we usually think of it as a disease accompanied by severe, lancinating pain, but this is not the case in cancer of the cervix. The cervix is by no means as sensitive as other portions of the body. As you know, we can hook tenacula in it, and even burn it with the hot iron, and cause little, if any, suffering. The disease may, at the beginning, be without pain, but as it advances to the internal os, pain is experienced, and when it involves the cavity of the womb, the woman suffers the tortures of the damned. I know nothing which equals the agonizing pain of cancer of the cavity of the uterus.

Having discovered a cancer, what do you tell the patient? In answering this question, I shall describe a case which I saw this morning. A lady was brought to my office by her physician, who suspected that she had a cancer of the cervix, but not being certain, desired my opinion. She was a stout woman, the mother of eleven children, ten of whom were living. Four years ago, after the birth of her last child, she began to lose a little more than usual, and soon she had a constant dribbling. She did not have the cancerous cachexia, and I therefore expected to find a polypus. As soon, however, as I examined her, I found that it was a cancer, the whole cervix being eaten away. I asked the lady to step into another room; I then told the physician what I had found, and he agreed with me that we should not tell her the exact nature of the trouble. We told her husband, however, that his wife had a cancer, and asked him if we should tell his wife. "No," he said, "do not tell her." Never say "cancer" to a woman, if you can possibly avoid it. You are signing her death warrant, and at once she gives up everything and goes down hill. I have had patients who, I am sure, knew what the disease was, and yet the word "cancer" never passed their lips. They always spoke of it as "the disease," or "the ulceration."

After talking the matter over with her doctor, I told her that she had a bad ulceration of the womb, and that we were going to help her as much as possible, but it was very hard to cure.

In reference to the treatment of this case, I said to the physician: "This woman has but a short time to live, and we must make the few remaining months of her life as comfortable as possible. Give her as much morphia as is necessary to relieve her pain. Give her a solution of morphia containing a quarter of a grain to the teaspoonful, and let her take it as she requires it." He objected to morphia in solution, because there were so many children about the house. We therefore substituted Schieffelin's granules, containing one-eighth of a grain of morphia, to be taken as often as was necessary. Morphia in solution is the best form of opium in these cases, because it is the cheapest. These patients must become opium eaters. Let the few days of their existence be free from pain. Give them a euthanasia, a pleasant death.

There was no use of attempting local treatment, but in order to build her up and retard the progress of the disease, I ordered her after meals a solution of the four chlorides:

℞ Hydrarg. Chloridi Corosivi, gr. $\frac{1}{24}$.
Liq. Arsenici Chloridi, gtt. ij.
Tinct. Ferri Chloridi.
Acidi Hydrochlorici Diluti, aa. gtt. x. M.

To check the dribbling, I ordered injections twice a day of strong cider vinegar, as hot as possible. If this failed, a saturated solution of alum is to be substituted. If both fail, then Monsel's solution (one part to five of water) will be employed. I do not like the latter injection because it causes plaster-like clots, which are apt to remain in the vagina, causing an offensive discharge.

Let us now turn to our patient. In order to show you the color of the skin I expose her breast. There is a sort of ashy, leaden hue, but it is not very marked. This hue is pathognomonic only after the cancer has become ulcerated. It is never seen in the scirrhus form. It is not due to the impregnation of the body with the cancer poison as such, but to the distribution of a poison from the local sore, septic in its character. This is shown by the fact that when these friable vegetations are removed, converting the cancer into a healthy sore, the cachectic appearance passes away and the natural color returns. You must not depend upon the color in the diagnosis of carcinoma, for I have seen cancer in women of a florid appearance. In such cases, my experience is that operation is of but little benefit.

There is a very bad odor from this vagina. My hand will be saturated. I should not like to perform an obstetric operation immediately after leaving this case. In order, however, to be prepared, I use only my left hand in gynecological operations, and reserve my right hand for obstetric manipulations. I am not fond of bad odors. I remember that our professor of practice used to say, when lecturing on diarrhœa and dysentery, that the stools of a patient ought to be as acceptable to a physician as a bouquet to a lady. I have never found it so, and have never been able to overcome an invincible aversion to foul odors. Permanganate of potassium is the best substance for removing this smell, but it stains the hands so that they are not presentable for several days. I shall therefore first wash them with soap and water, then with turpentine, which is a good antiseptic, again with soap and water, and lastly with carbolized water; but even after all this I do not expect to be entirely rid of the smell.

I shall now pass my finger into the vagina. This is a bad case. The posterior wall of the vagina is just about being attacked by the disease. The anterior lip of the cervix is comparatively healthy. I feel a notch posteriorly towards the right, where the laceration, which was the beginning of the disease, probably occurred. This would indicate an occipito-posterior position of the head. With this serrated curette, I shall remove as much as possible of this friable tissue. I find a projecting mass as large as a marble, which I twist off. The operation is not going to do this woman much good, as the disease is too far advanced.

The dangers from this operation are very slight. I know of no operation in which the after-history is so devoid of interest. Macaulay says: " Blessed is that nation which has no history," meaning that history is made up of a record of wars, acts of cruelty and injustice. In the same way, we may say, that these cases are blessed, inasmuch as their after-history contains no unfavorable symptoms. There is rarely pain or fever. I never had a fatal result until three months ago. I had been in consultation with a physician in the interior of the state in regard to a patient with cancer of the cervix. I was to go to perform the operation; the time was set and the physicians invited to attend, when I received a telegram telling me not to come. She had heard that clover tea was an excellent remedy for cancer, and her physician, very properly, knowing the incurable nature of the disease, gave her an opportunity of trying it. She used it for a month, but, finding herself no better, concluded to have the operation, and came to the city for that purpose. She arrived the day before I left on my vacation. I performed the operation. There was considerable hemorrhage which was readily checked. I left her in competent hands. Previous to coming here she had been allowed to have opium (which was entirely proper), and, thinking that we did not give her enough, got out of bed the night after the operation, and the succeeding night, and helped herself. This was followed by secondary hemorrhage, which carried her off. This is the only case of serious hemorrhage which I have met with after this operation, but where a cancerous cervix has been removed by the galvano-cautery, I have seen terrible hemorrhage and some deaths. One death occurred in my own practice and another in the practice of a friend. A third case of desperate hemorrhage occurred, in this building, in which life was barely saved. I shall never remove another cancerous cervix with the galvano-cautery. I very rarely use it for any purpose since the invention of Paquelin's thermo-cautery.

I have now removed a large amount of this friable tissue and can now insert four fingers into the cervix. Although, as I have said, this operation is not going to effect a cure, yet we are justified in performing it, for it will stop the hemorrhage, prolong her life and make her more comfortable. The older the woman, the better are the results from operation.

I should like to cauterize this with Paquelin's cautery, but, as our instrument is out of order, I shall employ nitric acid. By the way, let me say one word in regard to the use of the Paquelin's cautery. Never heat the platinum point to a white heat, for you are then liable to fuse the spongy platinum in the tip. If this is done, the instrument is useless. I know of no instrument maker in Philadelphia who can repair it.

I now have this as clean as possible; but here is something which I do not understand. I must examine and see where this little body comes from. Gentlemen, this is the second instance in which I have gotten into Douglas's cul-de-sac, in removing a carcinoma from the neck of the womb. This little body (about the size of a marble) which I show you is the hydatid of Morgagni. I do not like to get into the peritoneal cavity, but Carl Braun, of Vienna, in removing a cancerous cervix, does not fear getting into Douglas's pouch. He places the wire of the ecraseur sufficiently high to remove all the diseased tissue, without reference to the position of the peritoneum.

This makes the fourth time that I have got into the peritoneal cavity when I did not wish to. The first time was in removing the neck of the womb with the ecraseur. I made an opening as large as a silver quarter, but the woman recovered without a bad symptom. The second instance was exactly like the present one. Two or three years ago, while removing a cancer from the cervix by scraping, I made an opening into the peritoneum large enough to admit two fingers. This case worried me considerably, but she had not the slightest fever, and at the end of ten days went to her home, a distance of several hundred miles. The third case was one of vesico-vaginal fistula which had been accompanied by such inflammation and sloughing that I could not find the cervix and could not determine the position of the womb. In order to find the womb I operated, and while carefully dissecting upwards suddenly found myself in the cavity of the abdomen. I could, without trouble, feel the ovary. I immediately put her to bed. Her temperature ran up, but she recovered. I again operated with extreme care, but a second time I opened the peritoneum. This time she did not have an unfavorable symptom, although as she had a vesico-vaginal fistula the urine must, on both occasions, have found its way into the abdominal cavity. In this case I finally closed the vulva. The fourth case is the one before you.

Having made the opening, I shall examine the uterus. This I find to be enlarged, but the disease does not seem to have attacked it. I can readily feel the right ovary, and I think that I feel the top of the left ovary, but I shall not disturb the parts in order to settle this point. It is well that the woman is on her back, for if she were in Sim's, or the knee-breast position, the air would, at each inspiration and expiration, enter and be expelled from the peritoneal cavity; and I should not like the air from this room, which certainly is not very pure, to come in contact with the peritoneum.

I shall not make the application of nitric acid as I had intended, but shall thoroughly cleanse the vagina with carbolized water, put her to bed and give opium and quinia so as to be ready for any inflammation that may arise. If she were in a perfectly pure atmosphere, the danger would be very slight. The danger is from septic influences. I might say a word or two in regard to the hydatid of Morgagni. If you look in your dictionaries under this term, you will be referred to the corpora Morgagni, which are found in the testicle. The hydatid of Morgagni is a small body, a relic of fœtal life, which hangs from one of the fimbriæ of the fallopian tube, by a neat little stem about an inch long. The function of this little body we do not know. It is normally about the size of a pea, but it is sometimes enlarged. It is my opinion that some cases of bursting cyst of the abdomen, in which the cyst increases in size, then suddenly bursts and refills, are due to enlargement of the hydatid of Morgagni; and also that some of those cysts which have been described as cysts of the broad ligament with a pedicle are in reality cysts of this organ.

Gentlemen, I could have disguised from you the fact that I had opened Douglas's pouch, but I say that every one of us meets with mishaps, and that man is the best surgeon or the best physician who makes the fewest mistakes and meets with the fewest mishaps. If, in your practice, you should meet with this accident, it will afford you great comfort to be able to look back and say that "Dr. Goodell, with all his experience, did the same thing himself." This is a mishap, inasmuch as I should not have scraped so far back if I had thought the disease had advanced so near the peritoneum. Although I have the authority of Carl Braun for opening the peritoneal cavity, I do not agree with him as to its propriety, for the disease is a hopeless one. If by opening the peritoneum we could effect a cure, I should not hesitate. One object is not to shorten life, but to prolong it.*

### HYSTERICAL ENLARGEMENT OF THE ABDOMEN.

Our next patient is a woman 49 years of age. She has had six children, the youngest of which is sixteen years of age. She has had one miscarriage. The menopause occurred two years ago. Previously to the cessation of the menses, she had a severe attack of hæmatemesis. Since then she has had several attacks, none of which have been as severe as the first. These have occurred at what would have been the menstrual periods, if she had been menstruating. For the past two years, the abdomen has been enlarging, and she states that there is a body protruding from the vagina.

Women, at the change of life, are very liable to grow fat and to suffer from distension of the intestines, nervous flatulency, due to disturbances in the circulation of the nerve and blood fluids. As a result of this enlargement, especially if the menses have ceased, the woman often imagines that she is pregnant. When such cases come to you, a careful examination must be made, and it is sometimes difficult to make a positive diagnosis. So that in this respect an abdominal enlargement is often an abominable enlargement.

Here the abdomen is of large size. Pinching up its walls, I find that there is at least an inch and a half of fat. Beneath the fat, I feel a hard body, which may be due to rigidity of the muscles. I shall try percussion. This is of great service in determining the character of an abdominal enlargement. I find resonance all over. In the epigastrium, a little pain is caused by percussion.

I can say, with a great deal of confidence, that there is no solid tumor in the lower part of the abdomen, for we have re-

* The opening of the peritoneal cavity was followed by no elevation of temperature or other unfavorable symptom. The patient recovered.

sonance. A tumor of any size would push the intestines to one side, and there would be flatness, except where, as happens in rare instances, a coil of intestine gets in front of the tumor. There may possibly be fluid in the peritoneal cavity on which the intestines float.

You observe that while I have been talking she has lost some of her nervousness; she has been thrown off her guard and the abdomen is flattening down. I should like to examine her *per vaginam*, but as the hour has so nearly expired, I shall postpone that until she is removed; but I am disposed to think that it is nothing but an hysterical tumor, due to an accumulation of fat and flatus. I am not entirely satisfied in regard to the cause of the epigastric tenderness, and it may be necessary to examine her under ether. In the meantime, I shall give her bromide of potassium, which is our sheet-anchor in these cases.

This accumulation of fat in women is a curious affair. I should esteem it a great favor if some one would give me a prescription which would, in the first place, diminish the amount of fat, and in the second place remove certain ailments to which fat women are liable. Fat men suffer from the inconvenience and discomfort caused by the excess of fat, but they do not have the obscure troubles that women have. Fat women are liable to have peculiar phenomena referred to the uterine organs, the exact nature of which I am not able to explain. Whether they are due to a loss of retentive power, or whether the sagging over of the abdominal walls causes a suction power, drawing the womb up, I can not say. I have sometimes thought that the sagging down of the heavy abdominal walls tends to produce a vacuum, thus causing a drawing up of the pelvic organs. As a rule, in these cases we find the uterus high up.

If this patient will return next week, I shall examine her under ether.

